# Supplementary material for: Near real-time data on the human neutralizing antibody landscape to influenza virus as of early 2026 to inform vaccine-strain selection
Source: Virus Evol. 2026 Jul 26;12(1):veag046. doi: 10.1093/ve/veag046 (PMC13499528; doi:10.1093/ve/veag046)
Supplement: Supplementary file 1 [file supplementary_material__veag046.zip › supplementary-material.pdf]

Supplementary material

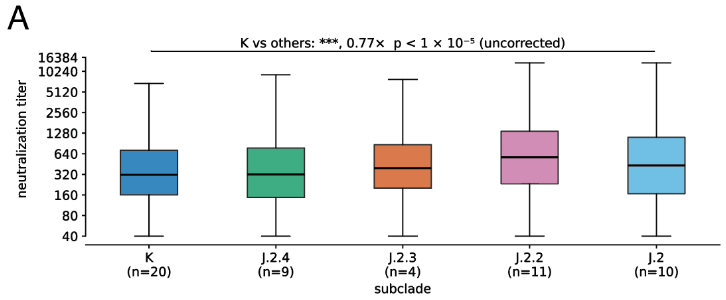

B

Comparison	Fold change	Sig.	p (BH)	p (raw)
J.2.2 vs. J.2	1.29×	***	$< 1\times10^{-5}$	$< 1\times10^{-5}$
J.2.3 vs. J.2	0.96×	ns	0.713	0.642
J.2.3 vs. J.2.2	0.75×	***	$< 1\times10^{-5}$	$< 1\times10^{-5}$
J.2.4 vs. J.2	0.80×	***	$< 1\times10^{-5}$	$< 1\times10^{-5}$
J.2.4 vs. J.2.2	0.62×	***	$< 1\times10^{-5}$	$< 1\times10^{-5}$
J.2.4 vs. J.2.3	0.82×	***	$< 1\times10^{-5}$	$< 1\times10^{-5}$
K vs. J.2	0.78×	***	$< 1\times10^{-5}$	$< 1\times10^{-5}$
K vs. J.2.2	0.61×	***	$< 1\times10^{-5}$	$< 1\times10^{-5}$
K vs. J.2.3	0.81×	***	$< 1\times10^{-5}$	$< 1\times10^{-5}$
K vs. J.2.4	0.98×	ns	0.920	0.920

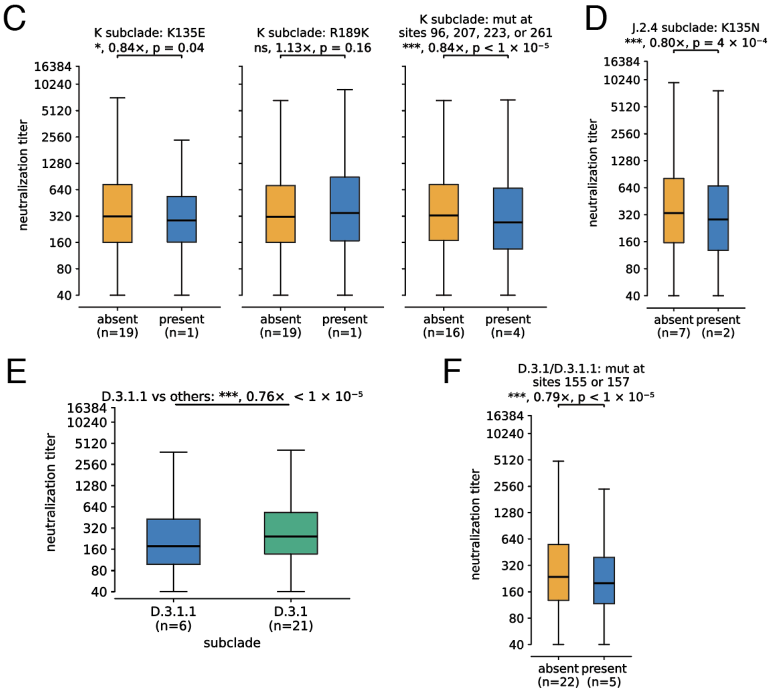

Supplementary Figure 1. Statistical analysis on aggregated titers measured from 302 human serum samples.

(A) Comparison of neutralization titers for all sera against all H3N2 subclade K viruses against all other 2025-2026-circulating strains in the library taken across all sera. The titers to K are significantly lower than the titers to other subclades (Mann-Whitney U test $p < 1e-5$). In the x-axis labels, n indicates the number of strains in that subclade in our library, and the box plots are the distribution of titers against those strains for all 302 sera. **(B)** All pair-wise comparisons between the different 2025-2026-circulating H3N2 subclades in the library with raw and Benjamini-Hochberg corrected p-values from the Mann-Whitney U test. Comparisons highlighted in green reached statistical significance. **(C)** Comparison of neutralization titers for all sera against all subclade K viruses with and without specific mutations or mutations at sets of sites in antigenic regions D and E, with Mann-Whitney p-values indicated. In the x-axis labels, n indicates the number of strains with that mutation. **(D)** The same as in **(C)**, but for subclade J.2.4 with and without K135N mutations. **(E)** The same as in **(B)**, but for H1N1 subclade D.3.1.1 against the other 2025-2026-circulating H1N1 subclade in the library. **(F)** The same as in **(C)** and **(D)**, but for H1N1 strains with and without mutations at sites 155/157.

Supplementary File 1. CSV with details about the strains in the viral library

Provides the strain name, collection date, subclade, derived haplotype, and nucleotide and protein HA ectodomain sequence for all viral strains in the library. Download or view at: https://github.com/jbloomlab/flu-seqneut-2025to2026/blob/main/results/final_titer_data/human_viruses.csv.

Supplementary File 2. CSV with details about the tested sera

Provides details about all sera assayed in the reported experiments. Download or view at: https://github.com/jbloomlab/flu-seqneut-2025to2026/blob/main/results/final_titer_data/human_sera.csv.

Supplementary File 3. CSV with all neutralization titers reported here

Provides neutralization titers measured by the reported experiments. Download or view at: https://github.com/jbloomlab/flu-seqneut-2025to2026/blob/main/results/final_titer_data/human_titers.csv.
